# Presence of autoantibodies in serum does not impact the occurrence of immune checkpoint inhibitor-induced hepatitis in a prospective cohort of cancer patients

**DOI:** 10.1007/s00432-021-03870-6

**Published:** 2021-12-07

**Authors:** Mette-Triin Purde, Rebekka Niederer, Nikolaus B. Wagner, Stefan Diem, Fiamma Berner, Omar Hasan Ali, Dorothea Hillmann, Irina Bergamin, Markus Joerger, Martin Risch, Christoph Niederhauser, Tobias L. Lenz, Martin Früh, Lorenz Risch, David Semela, Lukas Flatz

**Affiliations:** 1 Institute of Immunobiology, Kantonsspital St. Gallen, Rorschacher Strasse 95, 9007 St. Gallen, Switzerland; 2 Department of Dermatology, Venereology and Allergology, Kantonsspital St. Gallen, Rorschacher Strasse 95, 9007 St. Gallen, Switzerland; 3 Department of Oncology and Hematology, Kantonsspital St. Gallen, Rorschacher Strasse 95, 9007 St. Gallen, Switzerland; 4 Department of Oncology and Hematology, Hospital of Grabs, Spitalstrasse 44, 9472 Grabs, Switzerland; 5 Department of Dermatology, University Hospital Zurich, Rämistrasse 100, 8091 Zurich, Switzerland; 6 Labormedizinisches Zentrum Dr Risch Ostschweiz AG, Brauerstrasse 95, 9016 St. Gallen, Switzerland; 7 Department of Gastroenterology and Hepatology, Kantonsspital St. Gallen, Rorschacher Strasse 95, 9007 St. Gallen, Switzerland; 8 Interregional Blood Transfusion SRC, Murtenstrasse 137A, 3008 Bern, Switzerland; 9 University of Lausanne, 1015 Lausanne, Switzerland; 10 University of Bern, Hochschulstrasse 6, 3012 Bern, Switzerland; 11 Research Group for Evolutionary Immunogenomics, Max Planck Institute for Evolutionary Biology, August-Thienemann-Straße 2, 24306 Plön, Germany; 12 Department of Dermatology, University Hospital Tübingen, 72016 Tübingen, Germany

**Keywords:** Autoantibodies, Checkpoint inhibitors, Drug-induced liver injury, Drug-related side effects and adverse reactions

## Abstract

**Purpose:**

Immune checkpoint inhibitor (ICI)-induced hepatitis belongs to the frequently occurring immune-related adverse events (irAEs), particularly with the combination therapy involving ipilimumab and nivolumab. However, predisposing factors predicting the occurrence of ICI-induced hepatitis are barely known. We investigated the association of preexisting autoantibodies in the development of ICI-induced hepatitis in a prospective cohort of cancer patients.

**Methods:**

Data from a prospective biomarker cohort comprising melanoma and non-small cell lung cancer (NSCLC) patients were used to analyze the incidence of ICI-induced hepatitis, putatively associated factors, and outcome.

**Results:**

40 patients with melanoma and 91 patients with NSCLC received ICI between July 2016 and May 2019. 11 patients developed ICI-induced hepatitis (8.4%). Prior to treatment, 45.5% of patients in the hepatitis cohort and 43.8% of the control cohort showed elevated titers of autoantibodies commonly associated with autoimmune liver diseases (*p *= 0.82). We found two nominally significant associations between the occurrence of ICI-induced hepatitis and HLA alleles associated with autoimmune liver diseases among NSCLC patients. Of note, significantly more patients with ICI-induced hepatitis developed additional irAEs in other organs (*p *= 0.0001). Neither overall nor progression-free survival was affected in the hepatitis group.

**Conclusion:**

We found nominally significant associations of ICI-induced hepatitis with two HLA alleles. ICI-induced hepatitis showed no correlation with liver-specific autoantibodies, but frequently co-occurred with irAEs affecting other organs. Unlike other irAEs, ICI-induced hepatitis is not associated with a better prognosis.

**Supplementary Information:**

The online version contains supplementary material available at 10.1007/s00432-021-03870-6.

## Introduction

Immune checkpoint inhibitors (ICI) have significantly prolonged the survival of patients with various cancers (Iorgulescu et al. 2018; Massari et al. 2018). ICI are monoclonal antibodies that target inhibitory immune checkpoints on T cells, thereby reinvigorating their anti-tumor activity (Silva et al. 2018). Currently FDA-approved ICI target either programmed cell death protein 1 (PD-1), its ligand programmed death ligand 1 (PD-L1), or cytotoxic T lymphocyte-associated protein 4 (CTLA4).

The clinical use of ICI is curtailed by immune-related adverse events (irAEs) that develop in up to 70% of all patients (Freeman-Keller et al. 2016; Wang et al. 2017; Owen et al. 2018). Severe irAEs require therapy interruption or discontinuation and systemic immunosuppression, which may inhibit therapeutic efficacy (Brahmer et al. 2018).

ICI-induced hepatitis is relatively common but remains poorly characterized. Studies suggest that it occurs in up to 20% of patients, depending on the ICI treatment (Nishida and Kudo 2019). ICI-induced hepatitis is usually asymptomatic and detected by elevated liver enzymes (European Association for the Study of the Liver. Electronic address et al. 2019). The histopathological traits of ICI-induced hepatitis overlap with both viral and autoimmune hepatitis (Nadeau et al. 2018; Suzman et al. 2018).

Autoimmune liver diseases include autoimmune hepatitis (AIH), primary biliary cholangitis, and primary sclerosing cholangitis, as well as their overlap syndromes. All of these diseases are associated with various autoantibodies and human leukocyte antigen (HLA) alleles (Chen et al. 2019; Lee and Ronnekleiv-Kelly 2019).

The application of ICI in patients with preexisting liver diseases or hepatic dysfunction is controversial due to lacking reports (Kanz et al. 2016; Aizawa and Hokari 2017; Suzman et al. 2018; Kehl et al. 2019; Shah et al. 2019). As of now, there are no validated biomarkers for predicting the development of ICI-induced hepatitis. To better understand the development of ICI-induced hepatitis, we characterized it with clinical and laboratory parameters in a prospective cohort of metastatic cancer patients receiving ICI.

## Materials and methods

### Study population

We established a prospective observational cohort of metastatic melanoma and non-small cell lung cancer (NSCLC) patients that started treatment with ICI (anti-PD-1, anti-PD-L1, anti-CTLA4, or combination treatment). The study was conducted at the Kantonsspital St. Gallen, Switzerland. All participants provided written informed consent prior to study inclusion. We used the STROBE cohort checklist when writing our report (von Elm et al. 2014).

Patients were enrolled between July 15, 2016 and May 1, 2019. Follow-up data were collected until October 15, 2019. Patients without ICI-induced hepatitis who received at least five cycles of any ICI therapy were included in the control group. Patients without ICI-induced hepatitis who had received less than five cycles of ICI therapy were excluded from the analysis of putative predictive factors. None of the patients had preexisting autoimmune liver diseases. The frequency of other preexisting autoimmune diseases was comparable in patients with and without ICI-induced hepatitis.

The following clinical information was recorded for all participants: sex, age, preexisting autoimmune diseases, and previous cancer therapy. In case of early dropout or exclusion, timepoint of and reason for leaving the study were recorded. Response to ICI therapy was determined based on the first computed tomography (CT) scan at cycle 4–6 (8–12 weeks) and according to the RECIST criteria version 1.1 (Eisenhauer et al. 2009). The presence and grade of ICI-induced hepatitis were defined according to CTCAE version 5.0 (2017, European Association for the Study of the Liver. Electronic address et al. 2019). The grade was based on liver function tests [LFTs; AST, ALT, bilirubin, and alkaline phosphatase (ALP)], as previously defined (Brahmer et al. 2018). All LFTs were measured from before the first ICI administration until study end or dropout.

Two experienced board-certified gastroenterologists and hepatologists (IB and DS) reviewed all cases independently and determined the likelihood of ICI-induced hepatotoxicity versus an alternative etiology according to well-characterized criteria (Benichou et al. 1993; Fontana et al. 2009). The R value for liver injury was calculated by dividing the peak serum level of ALT/ULN by that of ALP/ULN (European Association for the Study of the Liver. Electronic address et al. 2019). The type of liver damage was determined from the R value as hepatocellular (*R* > 5), mixed (2 ≤ *R* ≤ 5), or cholestatic (*R* < 2) (European Association for the Study of the Liver. Electronic address et al. 2019).

Furthermore, we recorded the occurrence of irAEs affecting other organs, defined according to the CTCAE version 5.0 (2017, European Association for the Study of the Liver. Electronic address et al. 2019). The onset, duration, and type of systemic immunosuppressive therapy for ICI-induced hepatitis treatment, as well as the presence of metastases and other lesions in the liver (e.g., steatosis; based on ultrasonography and CT scans) were also recorded.

### Blood sampling and analyses

Blood sampling and processing was performed at every visit according to the treating physician’s instructions. In the hepatitis group, patient sera were analyzed at three timepoints: before first ICI application, at the onset of ICI-induced hepatitis (elevated LFTs), and 2 months later. For control patients without ICI-induced hepatitis, we analyzed pre-therapy serum samples.

We measured autoantibodies that have been associated with autoimmune liver diseases (listed in Supplementary Table 1). The titer and pattern of anti-nuclear (ANA) and anti-cytoplasmatic antibodies, as well as the titers of anti-smooth muscle, anti-actin, anti-mitochondrial, and anti-liver-kidney microsomal antibodies were determined by the indirect immunofluorescence test on the IF Sprinter (Euroimmun, Lübeck, Germany) according to the manufacturer’s instructions. ANA patterns were determined in adherence to the nomenclature of the International Consensus on ANA Patterns (Chan et al. 2015). Serum concentration of perinuclear anti-neutrophil cytoplasmatic antibodies was measured by fluorescent enzyme immunoassay on the Phadia 250 (Thermo Fisher Scientific, Waltham, MA). The following autoantibodies were detected via immunoblot analysis using the EUROLINE Autoimmune Liver Diseases Profile (Euroimmun, Lübeck, Germany): AMA-M2, M2-3E (BPO), Sp100, PML, gp210, LKM1, LC1, SLA/LP, and Ro-52. Total serum IgG was measured by turbidimetry on the Optilite (The Binding Site, Birmingham, UK).

Sera from hepatitis patients were screened for infection with hepatitis A, B, C, and E viruses (HAV, HBV, HCV, and HEV, respectively). HAV IgG and IgM, HCV IgG, HBV surface antigen and antibodies against HBV core antigen were detected by electrochemiluminescence immunoassay on the Cobas 6000 (Roche Diagnostics, Rotkreuz, Switzerland). HCV antigen was detected by chemiluminescent microparticle immunoassay on the Architect i2000SR (Abbott Laboratories, Lake Bluff, IL). HEV IgG and IgM were measured using enzyme-linked immunosorbent assays on the DSX (Dynex Technologies, Chantilly, VA). HEV PCR was performed on the Cobas 8800 (Roche Diagnostics, Rotkreuz, Switzerland). The inter-assay coefficients of variation for relevant analyses are provided in Supplementary Table 2.

### HLA typing

DNA for HLA haplotyping was isolated from peripheral blood mononuclear cells. All patients were genotyped for HLA loci A, B, C, DPB1, DQB1, and DRB1. Genotyping was performed using next-generation sequencing at six-digit resolution (4 × High Resolution Typing, HistoGenetics, Ossining, NY).

### Statistical analysis

Continuous variables were compared using unpaired two-tailed *t* test with Welch’s correction. Proportions were compared using Chi-squared test with or without Yates’ correction, as appropriate. Patients whose response to ICI therapy at first CT scan was unknown were excluded from analyses pertaining to therapy response. Survival curves were compared by log-rank test. Patients who were alive or progression-free at the end of the follow-up period were censored in overall and progression-free survival analysis, respectively. Patients lost to follow-up were censored at the timepoint of last contact. To correct for multiple comparisons of survival curves, p values were adjusted using the Holm–Šídák method. All statistical analyses were performed using Prism version 8.1.2 (GraphPad Software, San Diego, CA).

## Results

### Patient characteristics

135 patients were screened for study inclusion. Four patients were excluded: three because they never began ICI therapy and one due to change of therapy after one cycle of ICI treatment (Supplementary Fig. 1). We included 131 patients, of whom 11 developed ICI-induced hepatitis (8.4%). The average onset of ICI-induced hepatitis was 12.8 weeks (range 2.7–48.9 weeks) after therapy initiation. 73 patients fulfilled the inclusion criteria for the control group. The mean follow-up time was 53.4 weeks (1–153 weeks). Detailed clinical characteristics of the hepatitis patients are shown in Table 1.

**Table 1:** Clinical characteristics of patients with ICI-induced hepatitis

Patient	Tumor	ICI target	ICI response	Hepatitis grade (AST/ALT)	Bilirubin elevation grade	Onset (days)	*R* value	Liver damage	Steroids duration (days)	Other irAEs	Liver metastasis	Other preexisting liver issues	Comments
1	NSCLC	PD-1	PD	2	2	27	0.78	Cholestatic	13	–	–	–	
2	NSCLC	PD-1	SD	2, 1	0, 1	342	0.66	Cholestatic	–	Skin	+	–	Preexisting LFT elevations (medication)
3	Melanoma	CTLA4	PD	3	0	42	2.86	Mixed	–	GIT, PNS	+	–	Liver metastasis too small to explain grade 3 hepatitis
4	NSCLC	PD-1	PR	2, 1	1, 0	155	1.60	Cholestatic	–	Skin	–	steatosis	Preexisting LFT elevations (medication)
5	NSCLC	PD-1	SD	2	0	70	0.46	Cholestatic	–	Skin, endocrine, kidney	–	–	
6	Melanoma (uveal)	PD-1, CTLA4	PD	3	2	21	4.84	Mixed	146	Skin	+	–	
7	NSCLC	PD-L1	PD	2	0	19	1.63	Cholestatic	–	–	+	–	Small peripheral liver metastasis; cannot explain LFT elevations
8	Melanoma	PD-1	PR	3	0	28	15.47	Hepatocellular	7	GIT	Suspected (small)	Steatosis	
9	NSCLC	PD-1	PR	1	0	211	2.11	Mixed	30	Skin, GIT, lung	–	Steatosis	Preexisting diabetes type I
10	Melanoma	PD-1, CTLA4	CR	3	0	21	3.22	Mixed	145	Skin	–	–	LFTs rebounded twice to steroids
11	Melanoma	PD-1, CTLA4	PD	4	2	47	23.47	Hepatocellular	130	Skin	–	Liver cysts	

The type of liver damage was derived from the *R* value

*ALT* alanine transaminase, *AST* aspartate transaminase, *CR* complete response, *CTLA4* cytotoxic T lymphocyte-associated protein 4, *GIT* gastrointestinal tract, *ICI* immune checkpoint inhibitors, *irAE* immune-related adverse event, *LFT *liver function test, *NSCLC* non-small cell lung cancer, *PD* progressive disease, *PD-1* programmed cell death protein 1, *PD-L1* programmed death ligand 1, *PNS* peripheral nervous system, *PR* partial response, *SD* stable disease

Five patients (45.5%) presented with hepatitis of grade 3 or higher (Table 1). There were two cases of hepatocellular damage (*R* value = 15.47 and 23.47, respectively), while the other patients had liver damage of cholestatic or mixed type.

ICI therapy was stopped in nine patients and restarted in three of them after 2–7 weeks. In one patient, LFTs increased after another ICI dose and therapy was discontinued. Six patients received systemic steroid treatment, which led to the normalization of their LFTs after 1 week to several months (Table 1). In the remaining five patients, LFTs normalized without steroid use. No other immunosuppressants were given.

### Viral hepatitis

No evidence of an active infection with HAV, HBV, HCV, or HEV was found among the ICI-induced hepatitis group (Supplementary Table 3). Elevated HAV and HEV IgG indicated past infection with or vaccination against hepatitis A and/or E in five patients. Active infection was ruled out via negative IgM and viral load PCR.

### Autoantibodies associated with autoimmune liver diseases

We analyzed patient sera for the presence of autoantibodies associated with autoimmune liver diseases (listed in Supplementary Table 1). In 7/11 (63.6%) patients with ICI-induced hepatitis, we measured at least one elevated autoantibody (Supplementary Table 4). 5/11 (45.5%) hepatitis patients and 32/73 (43.8%) control patients had an undoubtedly positive titer of at least one autoantibody (*p *= 0.82; Table 2). When borderline positive titers were included, there was a higher proportion of patients with autoantibodies in the control group (82.2% vs 63.6%, *p *= 0.31). We observed no significant changes in the titers over the course of ICI treatment (Supplementary Table 4). We did not find statistically significant differences in ANA patterns or single antibodies between the two groups (Supplementary Table 5).

**Table 2:** Positive titers of liver autoantibodies in patients with and without ICI-induced hepatitis

	Number	Proportion
Hepatitis (*N* = 11)	5	45.5%
No hepatitis (*N* = 73)	32	43.8%
*p* value	0.82	

*ICI* immune checkpoint inhibitors

### HLA alleles

**Fig. 1:**
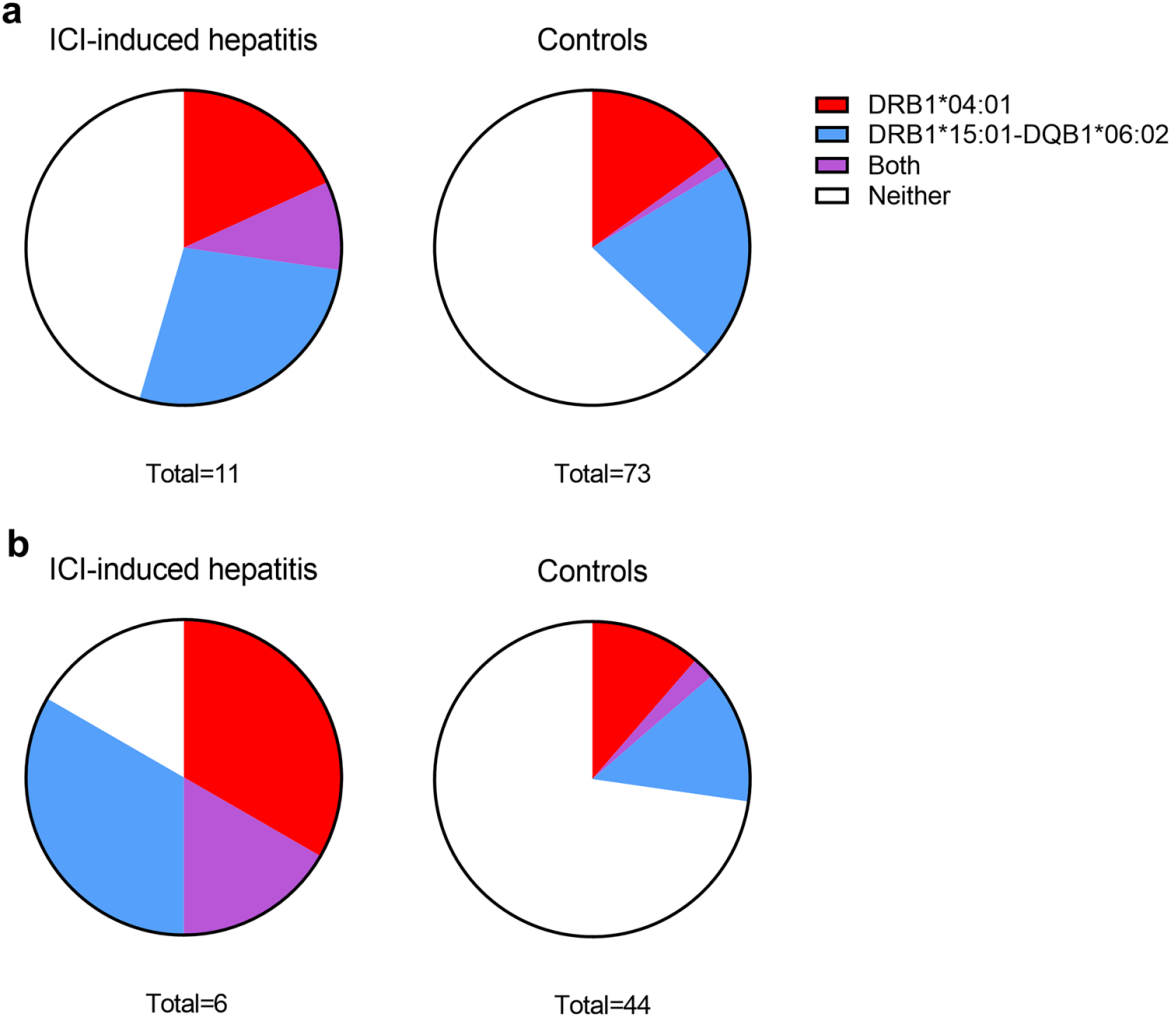
Proportion of HLA allele DRB1*04:01 and haplotype DRB1*15:01–DQB1*06:02 in patients with ICI-induced hepatitis and controls among the whole cohort (**a**) and among patients with NSCLC (**b**). These alleles are associated with autoimmune liver diseases and exhibited a significant association with ICI-induced hepatitis among NSCLC patients in this cohort. *ICI* immune checkpoint inhibitors, *NSCLC* non-small cell lung cancer

To assess the association between HLA and ICI-induced hepatitis, we screened hepatitis and control patients for the HLA alleles that are associated with a predisposition to or protection from autoimmune liver diseases (Supplementary Table 6). Among patients with NSCLC, two alleles were nominally significantly associated with the risk of developing ICI-induced hepatitis: DRB1*04:01 (*p *= 0.037) and the haplotype DRB1*15:01–DQB1*06:02 (*p *= 0.04) (Fig. 1). However, the statistical significance of those associations was absent in the whole patient population and after correcting for multiple comparisons, which indicates the need for studies in larger cohorts to confirm the finding.

### Survival and therapy response

In the group with ICI-induced hepatitis, there was one death during the study for medical reasons unrelated to the liver. There were no deaths caused by liver failure or liver metastasis.

**Fig. 2:**
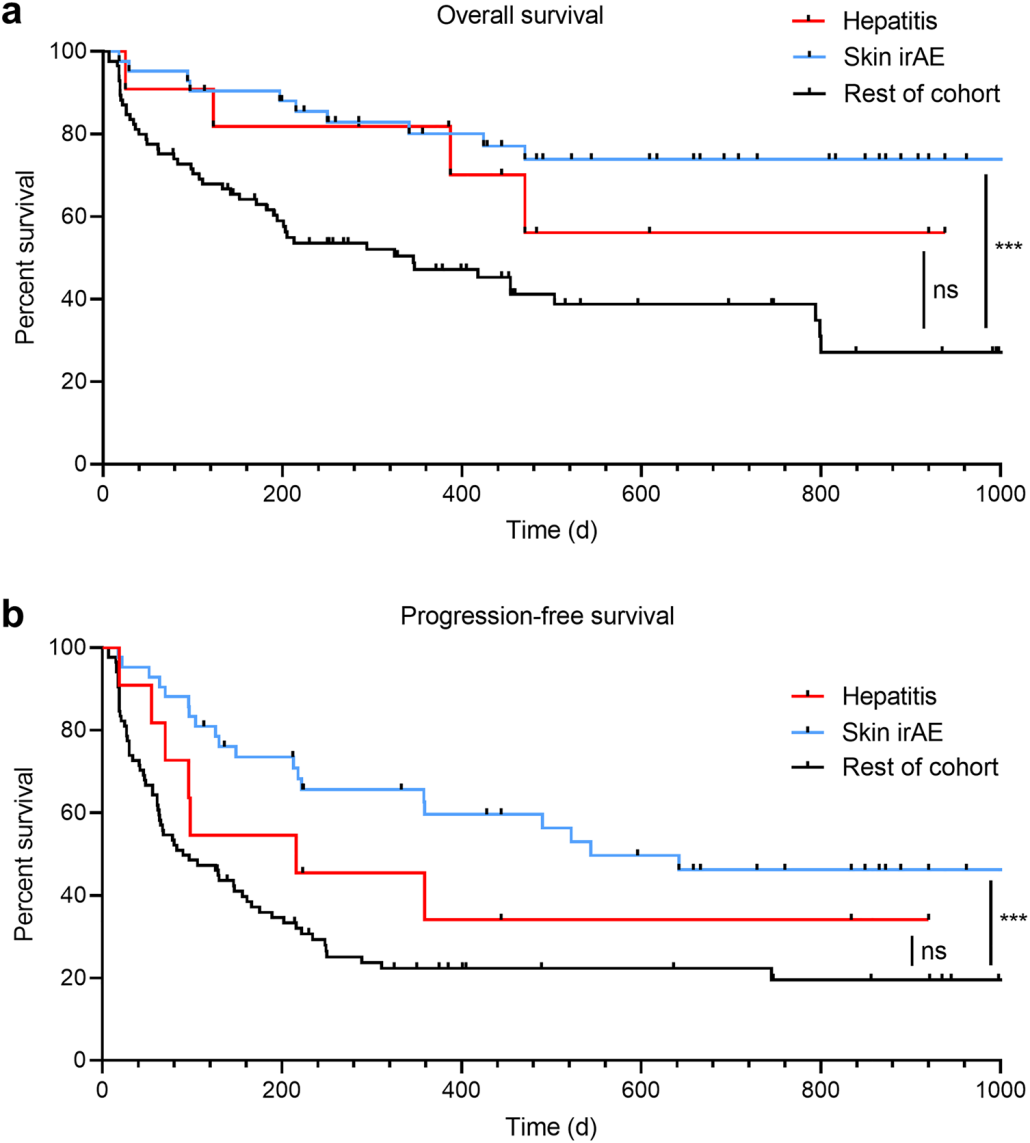
Overall (**a**) and progression-free survival (**b**) of patients with ICI-induced hepatitis and skin irAEs compared with the rest of the cohort. *d* days, *ICI* immune checkpoint inhibitors, *irAE* immune-related adverse event

IrAEs affecting the skin are well-known adverse effects of ICI therapy and associated with improved survival (Hasan Ali et al. 2016). 42 patients (32.1% of all patients) experienced skin irAEs in this study. We investigated the associations of overall survival (OS) and progression-free survival (PFS) with ICI-induced hepatitis and skin irAEs (Fig. 2). Only skin irAEs were associated with longer OS and PFS (*p *= 0.0003 for both). In contrast, there were no significant differences between patients with ICI-induced hepatitis and the rest of the cohort (OS, *p *= 0.23; PFS, *p *= 0.35). The patients with ICI-induced hepatitis did not respond more often to ICI therapy than the control group (36.4% vs 41.8%, *p *= 0.99). The proportion of patients with progressive disease was higher among patients with ICI-induced hepatitis (45.5% vs 13.4%, *p *= 0.03).

The patients with and without ICI-induced hepatitis did not differ significantly with regards to age, sex ratio, or prior exposure to ICI therapy (Table 3). One patient in the hepatitis group and three control patients had preexisting autoimmune diseases (type I diabetes mellitus, multiple sclerosis, Guillain–Barré syndrome, and ulcerative colitis). The proportion of patients with autoimmune diseases did not differ between the groups. However, we found a highly significant difference in the development of multiple irAEs: 9/11 patients in the ICI-induced hepatitis group (81.8%) developed additional irAEs, compared with 15/73 controls with multiple irAEs (20.5%; *p *= 0.0001).

**Table 3:** Comparison of clinical characteristics of patients with and without ICI-induced hepatitis

	ICI-induced hepatitis *N* = 11	Control group *N* = 73	*p* value
Age, median (range)	61 (41–73)	67 (47–86)	0.13
Sex, M:F	5:6	40:33	0.80
Prior ICI therapy, *N* (%)	2 (18.2%)	8 (11.0%)	0.85
Preexisting autoimmune disease, *N* (%)	1 (9.1%)	3 (4.1%)	0.97
Multiple irAEs, *N* (%)	9 (81.8%)	15 (20.5%)	**0.0001**

Bold value indicates statistically significant *p* value

*ICI* immune checkpoint inhibitor, *irAE* immune-related adverse event

## Discussion

This study systematically investigated the development of ICI-induced hepatitis during ICI therapy and their associations with patient characteristics. To this end, we established a prospective cohort of metastatic melanoma and NSCLC patients treated with ICI. Viral hepatitis was excluded in all patients with elevated LFTs. The incidence and average onset of ICI-induced hepatitis were comparable with previous publications (Karamchandani and Chetty 2018; Suzman et al. 2018).

It is known that liver metastases can increase LFTs (Beck et al. 1979; Cao and Wang 2012). In a recent study of liver injury in patients receiving pembrolizumab, pre-treatment liver metastases were identified as the only independent predictor of LFT elevations (Tsung et al. 2019). In our study, two patients (Patients 6 and 8) had LFT elevations that may have occurred due to a liver metastasis (Table 1). Two other patients (2 and 4) had elevated LFTs already prior to the start of ICI therapy, which we consider likely to be caused by other concomitant drugs.

We measured the titers of autoantibodies associated with classical autoimmune liver diseases in patients developing ICI-induced hepatitis. The data show no statistically significant differences in overall titers or specific autoantibodies compared with control patients. While other authors have shown associations between autoantibodies and the occurrence of ICI-induced skin toxicity, hypophysitis, and pneumonitis (Ali et al. 2019; Tahir et al. 2019), we did not find such an association with ICI-induced hepatitis. Our data are in line with the previous findings that show high levels of autoantibodies in healthy volunteers (Okamoto et al. 2004; Slight-Webb et al. 2016) and cancer patients (Anderson et al. 2015; Nisihara et al. 2018). Furthermore, the previous reports similarly show no elevated titers of liver-specific autoantibodies in patients with ICI-induced hepatitis (Johncilla et al. 2015; De Martin et al. 2018; Zen and Yeh 2018). Therefore, our data indicate that liver autoantibodies are not suitable predictors for the development of ICI-induced hepatitis. Moreover, this suggests that the pathogenesis of ICI-induced hepatitis may differ from that of classical autoimmune hepatitis. This also indicates that patients with preexisting liver autoantibodies do not have an increased risk of developing hepatitis during ICI treatment.

We studied whether HLA alleles associated with autoimmune liver diseases are also associated with ICI-induced hepatitis. Interestingly, we detected two alleles with nominal statistical significance among NSCLC patients. However, the significance disappeared upon correction for multiple comparisons, possibly because of the small sample size. The effect may have been too weak to detect at this study power. This highlights the necessity of conducting larger studies to investigate the role of HLA alleles in the development of ICI-induced hepatitis.

The occurrence of irAEs has been associated with better therapy response and prolonged survival (Sato et al. 2018; Okada et al. 2019). We observed this association for patients with skin irAEs. Conversely, the development of ICI-induced hepatitis did not correlate with OS or PFS and even showed an association with worse therapy response. This is in line with a recent study showing a trend toward favorable OS and PFS only for patients with ICI-induced hepatitis of grade 3–4 (Biewenga et al. 2021).

We also found a highly significant co-occurrence of ICI-induced hepatitis and other irAEs. However, patients with probable ICI-induced hepatitis have shown better survival than patients with liver injury due to other causes (Tsung et al. 2019) and a recent study showed improved overall survival in melanoma patients who developed elevated levels of gamma-glutamyl transferase during ICI treatment (Winter et al. 2021). It is possible that improved survival among the patients with ICI-induced hepatitis could not be detected in this study because of the small sample size.

Our study was limited by the small sample size of the ICI-induced hepatitis cohort, which may restrict statistical power. Nevertheless, we were able to show nominally significant associations between ICI-induced hepatitis and two HLA alleles, as well as a highly significant co-occurrence of ICI-induced hepatitis with other irAEs. Investigating these findings in larger cohorts may reveal additional associations. In a follow-up study, it would be beneficial to obtain liver biopsies from all patients with ICI-induced hepatitis to also include histological findings.

In conclusion, this study characterized ICI-induced hepatitis in detail in a prospective cohort of cancer patients receiving ICI treatment. Active hepatitis virus infection could be excluded in all patients with elevated LFTs. We found no associations between ICI-induced hepatitis occurrence and markers of classical autoimmune liver disease, suggesting a mechanistic difference in pathogenesis and no increased risk of ICI-induced hepatitis for patients with preexisting liver autoantibodies. In contrast, we discovered a nominally significant association between ICI-induced hepatitis and two HLA alleles in NSCLC patients. These data emphasize the role of genetic background as a major predictor of irAE development. Unlike skin irAEs, ICI-induced hepatitis was not associated with improved therapy response or survival, but was associated with additional irAEs. This implies that liver involvement may signal the presence of other irAEs.

## Supplementary Information

Below is the link to the electronic supplementary material.

Supplementary file1 (PDF 170 KB)

Supplementary file2 (PDF 67 KB)

Supplementary file3 (PDF 67 KB)

Supplementary file4 (PDF 80 KB)

Supplementary file5 (PDF 535 KB)

Supplementary file6 (PDF 359 KB)

Supplementary file7 (PDF 370 KB)

## Data Availability

The datasets used in this study are available from the corresponding author on reasonable request.
