# Supplementary material for: Presence of autoantibodies in serum does not impact the occurrence of immune checkpoint inhibitor-induced hepatitis in a prospective cohort of cancer patients
Source: J Cancer Res Clin Oncol. 2021 Dec 7;148(3):647–56. doi: 10.1007/s00432-021-03870-6 (PMC8881258; doi:10.1007/s00432-021-03870-6)
Supplement: Supplementary file 1 — Supplementary file1 (PDF 170 KB) [file 432_2021_3870_MOESM1_ESM.pdf]

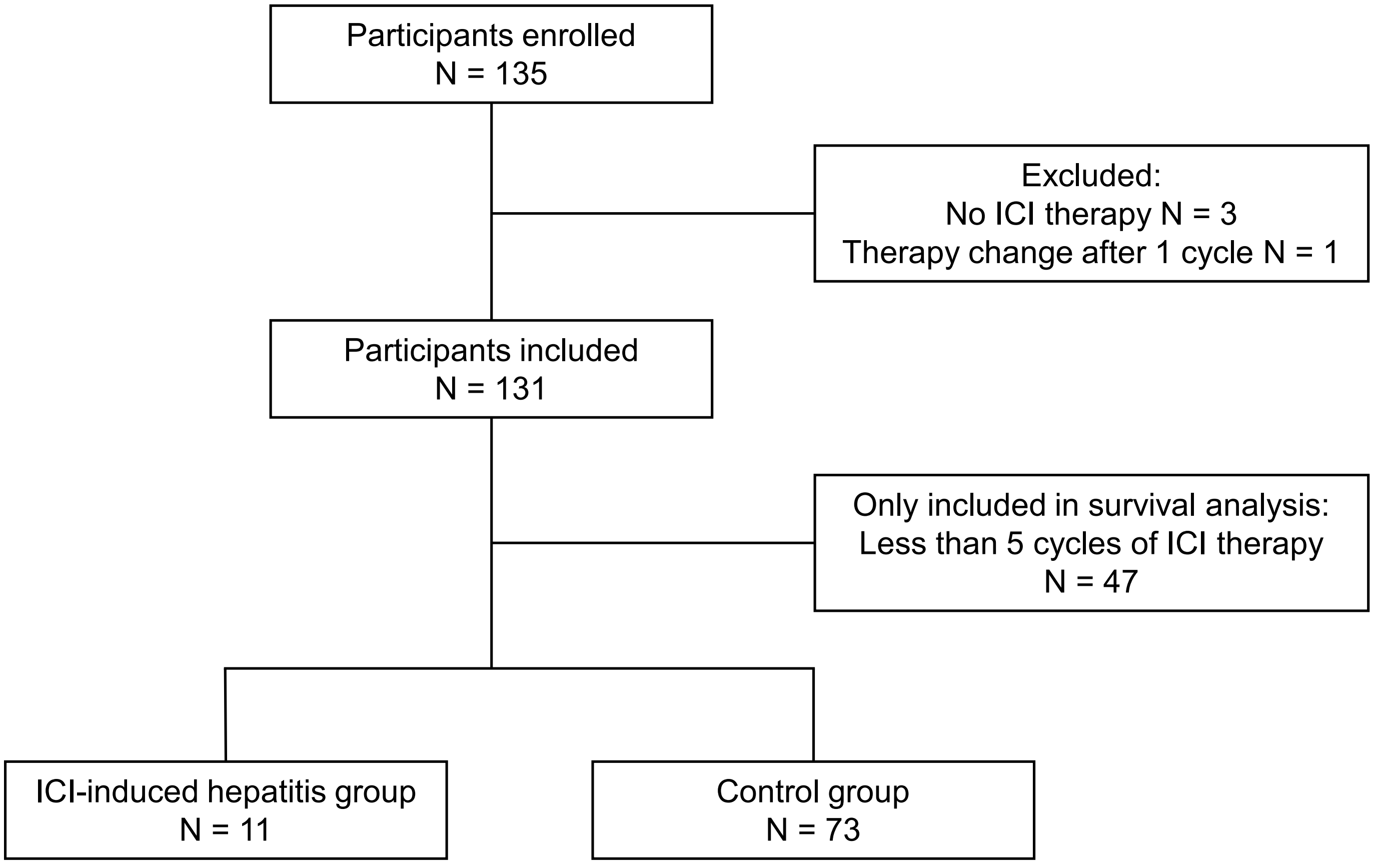

Supplementary Figure 1. Flowchart of participants in the study. ICI – immune checkpoint inhibitor.
