# Supplementary material for: Presence of autoantibodies in serum does not impact the occurrence of immune checkpoint inhibitor-induced hepatitis in a prospective cohort of cancer patients
Source: J Cancer Res Clin Oncol. 2021 Dec 7;148(3):647–56. doi: 10.1007/s00432-021-03870-6 (PMC8881258; doi:10.1007/s00432-021-03870-6)
Supplement: Supplementary file 2 — Supplementary file2 (PDF 67 KB) [file 432_2021_3870_MOESM2_ESM.pdf]

Supplementary Table 1. The autoantibodies analyzed in this study and their associated autoimmune liver diseases. AIH – autoimmune hepatitis, AMA – anti-mitochondrial antibody, ANA – antinuclear antibody, ASMA – anti-smooth muscle antibody, LKM – anti-liver-kidney microsomal antibody, pANCA – perinuclear anti-neutrophil cytoplasmatic antibody, PBC – primary biliary cholangitis, PSC – primary sclerosing cholangitis.

**Autoantibody**	**Associated liver disease(s)**	**References**
pANCA	AIH, PSC	[1]
Anti-actin	AIH	[2, 3]
ASMA	AIH	[2, 3]
AMA	PBC	[4, 5]
LKM	AIH	[3]
ANA	AIH, PBC	[3]

[1] Bansi D, Chapman R, Fleming K. Antineutrophil cytoplasmic antibodies in chronic liver diseases: prevalence, titre, specificity and IgG subclass. Journal of hepatology. 1996;24:581-6.

[2] Couto CA, Bittencourt PL, Porta G, Abrantes-Lemos CP, Carrilho FJ, Guardia BD, et al. Antismooth muscle and antiactin antibodies are indirect markers of histological and biochemical activity of autoimmune hepatitis. Hepatology. 2014;59:592-600.

[3] Toh BH. Diagnostic autoantibodies for autoimmune liver diseases. Clinical & translational immunology. 2017;6:e139.

[4] Hu CJ, Zhang FC, Li YZ, Zhang X. Primary biliary cirrhosis: what do autoantibodies tell us? World journal of gastroenterology. 2010;16:3616-29.

[5] Nakamura M. Clinical significance of autoantibodies in primary biliary cirrhosis. Seminars in liver disease. 2014;34:334-40.
