# Supplementary material for: Presence of autoantibodies in serum does not impact the occurrence of immune checkpoint inhibitor-induced hepatitis in a prospective cohort of cancer patients
Source: J Cancer Res Clin Oncol. 2021 Dec 7;148(3):647–56. doi: 10.1007/s00432-021-03870-6 (PMC8881258; doi:10.1007/s00432-021-03870-6)
Supplement: Supplementary file 3 — Supplementary file3 (PDF 67 KB) [file 432_2021_3870_MOESM3_ESM.pdf]

Supplementary Table 2. Inter-assay coefficient of variation for laboratory analyses. HAV – hepatitis A, HBV – hepatitis B, HCV – hepatitis C, Ig – immunoglobulin, pANCA – peri-nuclear anti-neutrophil cytoplasmatic antibody.

**Analysis**	**N 1**	**Mean 1**	**CV 1**	**N 2**	**Mean 2**	**CV 2**
Total IgG	113	31.39	4.26	113	87.81	3.26
HAV IgG/IgM	58	20.53	4.72	58	31.6	5.86
HAV IgM	76	0.33	2.4	264	2.01	9.68
HBV surface antigen	73	0.448	10.57	155	2.68	5.03
Anti-HBV core antigen	136	0.305	9.69	79	2.28	2.23
HCV IgG	358	3.27	6.21	360	0.06	10.13
HCV antigen	16	51.32	2.42	16	297.5	2.36
pANCA	14	19.57	9.96	14	21.36	10.33
