# Supplementary material for: Presence of autoantibodies in serum does not impact the occurrence of immune checkpoint inhibitor-induced hepatitis in a prospective cohort of cancer patients
Source: J Cancer Res Clin Oncol. 2021 Dec 7;148(3):647–56. doi: 10.1007/s00432-021-03870-6 (PMC8881258; doi:10.1007/s00432-021-03870-6)
Supplement: Supplementary file 4 — Supplementary file4 (PDF 80 KB) [file 432_2021_3870_MOESM4_ESM.pdf]

Supplementary Table 3. Analyses for hepatitis viruses in patients with elevated liver function tests. Baseline refers to the blood sample taken before the start of immunotherapy. Onset refers to the elevation of liver function tests. HAV – hepatitis A, HBcAg – antibodies against hepatitis B core antigen, HBsAg – hepatitis B surface antigen, HBV – hepatitis B, HCV – hepatitis C, HEV – hepatitis E, mo – months, n.d. – not detected.

		HAV		HBV		HCV		HEV				
Patient	Timepoint	IgG/IgM	IgM	HBcAg	HBsAg	IgG	Antigen	Screen	IgG	IgM	PCR	Conclusion
**1**	Baseline	3.69		n.d.	0.44	n.d.	< 3.0	n.d.			n.d.	No exposure
	Onset	4.59		n.d.	0.38	n.d.	< 3.0	n.d.			n.d.	
**2**	Baseline	**> 50**	n.d.	n.d.	0.34	n.d.	< 3.0	+	**8.6**	n.d.	n.d.	Past infection/ vaccination
	Onset	**> 50**	n.d.	n.d.	0.34	n.d.	< 3.0	+	**9.4**	n.d.	n.d.	
**3**	Baseline	< 3.0		n.d.	0.36	n.d.	< 3.0	n.d.			n.d.	No exposure
**4**	Baseline	3.79		n.d.	0.34	n.d.	< 3.0	n.d.			n.d.	No exposure
	Onset	4.24		n.d.	0.36	n.d.	< 3.0	n.d.			n.d.	
	Onset +2 mo	5.82		n.d.	0.37	n.d.	< 3.0	n.d.			n.d.	
**5**	Baseline	< 3.0		n.d.	0.28	n.d.	< 3.0	n.d.			n.d.	No exposure
	Onset	< 3.0		n.d.	0.42	n.d.	< 3.0	n.d.			n.d.	
	Onset +2 mo	< 3.0		n.d.	0.37	n.d.	< 3.0	n.d.			n.d.	
**6**	Baseline	< 3.0		n.d.	0.4	n.d.	< 3.0	n.d.			n.d.	No exposure
	Onset	3.06		n.d.	0.43	n.d.	< 3.0	n.d.			n.d.	
	Onset +2 mo	3.85		n.d.	0.42	n.d.	< 3.0	n.d.			n.d.	
**7**	Baseline	**> 50**	n.d.	n.d.	0.41	n.d.	< 3.0	n.d.			n.d.	Past infection/ vaccination
**8**	Baseline	**32.1**	n.d.	n.d.	0.34	n.d.	< 3.0	n.d.			n.d.	Past infection/ vaccination
	Onset	**35.24**	n.d.	n.d.	0.33	n.d.	< 3.0	n.d.			n.d.	
	Onset +2 mo	**31.77**	n.d.	n.d.	0.36	n.d.	< 3.0	n.d.			n.d.	
**9**	Baseline	15.54		n.d.	0.4	n.d.	< 3.0	n.d.			n.d.	No exposure
**10**	Baseline	**> 50**	n.d.	n.d.	0.37	n.d.	< 3.0	n.d.			n.d.	Past infection/ vaccination
	Onset	**> 50**	n.d.	n.d.	0.34	n.d.	< 3.0	n.d.			n.d.	
	Onset +2 mo	**> 50**	n.d.	n.d.	0.39	n.d.	< 3.0	n.d.			n.d.	
**11**	Baseline	**> 50**	n.d.	n.d.	0.38	n.d.	< 3.0	+	**3**	n.d.	n.d.	Past infection/ vaccination
	Onset	**> 50**	n.d.	n.d.	0.44	n.d.	< 3.0	+	**2.9**	n.d.	n.d.	
Reference range		< 20			< 1		< 3.0		< 0.8			
