# Supplementary material for: Presence of autoantibodies in serum does not impact the occurrence of immune checkpoint inhibitor-induced hepatitis in a prospective cohort of cancer patients
Source: J Cancer Res Clin Oncol. 2021 Dec 7;148(3):647–56. doi: 10.1007/s00432-021-03870-6 (PMC8881258; doi:10.1007/s00432-021-03870-6)
Supplement: Supplementary file 5 — Supplementary file5 (PDF 535 KB) [file 432_2021_3870_MOESM5_ESM.pdf]

Supplementary Table 4. Autoimmune liver disease-associated autoantibodies in patients with ICI-induced hepatitis. Onset refers to the elevation of liver function tests. Non-negative titers are indicated in bold. Some samples were not available due to patient loss to follow-up (withdrawal from the study due to disease progression or irAEs, patient death). ICI – immune checkpoint inhibitors, irAE – immune-related adverse event, mo – months, N/A – sample not available, neg – negative.

		Immunofluorescence		Immunoblot		
Patient	Timepoint	Nuclear	Cytoplasmic	Sp100	gp210	Ro-52
1	Pre-treatment	< 1:80	**1:160**	neg	neg	neg
	Onset	< 1:80	**1:80**	neg	neg	neg
	Onset +2 mo	N/A				
2	Pre-treatment	< 1:80	**1:80**	neg	neg	neg
	Onset	< 1:80	**1:80**	neg	neg	neg
	Onset +2 mo	N/A				
3	Pre-treatment	< 1:80	< 1:80	neg	neg	neg
	Onset	N/A				
	Onset +2 mo	N/A				
4	Pre-treatment	**1:80**	**1:80**	neg	neg	**++**
	Onset	< 1:80	**1:80**	neg	neg	**+++**
	Onset +2 mo	**1:320**	**1:80**	neg	neg	**+++**
5	Pre-treatment	**> 1:1280**	**1:80**	**++**	neg	neg
	Onset	**> 1:1280**	**1:80**	**++**	**(+)**	neg
	Onset +2 mo	**> 1:1280**	**1:80**	**++**	neg	neg
6	Pre-treatment	< 1:80	< 1:80	neg	**(+)**	neg
	Onset	< 1:80	**1:80**	neg	neg	neg
	Onset +2 mo	< 1:80	< 1:80	neg	neg	neg
7	Pre-treatment	< 1:80	< 1:80	neg	neg	neg
	Onset	N/A				
	Onset +2 mo	N/A				
8	Pre-treatment	**1:80**	< 1:80	neg	neg	neg
	Onset	**1:80**	< 1:80	neg	neg	neg
	Onset +2 mo	**1:80**	< 1:80	neg	neg	neg
9	Pre-treatment	**1:320**	< 1:80	neg	neg	**+++**
	Onset	N/A				
	Onset +2 mo	N/A				
10	Pre-treatment	< 1:80	< 1:80	neg	neg	neg
	Onset	< 1:80	< 1:80	neg	neg	neg
	Onset +2 mo	< 1:80	< 1:80	neg	neg	neg
11	Pre-treatment	< 1:80	< 1:80	neg	neg	neg
	Onset	< 1:80	< 1:80	neg	neg	neg
	Onset +2 mo	N/A				
Reference range		< 1:80	< 1:80			
