# Supplementary material for: Presence of autoantibodies in serum does not impact the occurrence of immune checkpoint inhibitor-induced hepatitis in a prospective cohort of cancer patients
Source: J Cancer Res Clin Oncol. 2021 Dec 7;148(3):647–56. doi: 10.1007/s00432-021-03870-6 (PMC8881258; doi:10.1007/s00432-021-03870-6)
Supplement: Supplementary file 6 — Supplementary file6 (PDF 359 KB) [file 432_2021_3870_MOESM6_ESM.pdf]

Supplementary Table 5. Proportion of positive autoantibody titers in patients with and without ICI-induced hepatitis (single antibodies and ANA patterns). For IgG, the mean value (in g/l) of both groups is given with the 95% confidence interval. p values above 0.05 are given as not significant (ns). ANA – anti-nuclear antibody, AMA – anti-mitochondrial antibody, ASMA – anti-smooth muscle antibody, IB – immunoblot, ICI – immune checkpoint inhibitors, Ig – immunoglobulin, LKM – anti-liver-kidney microsomal antibody, pANCA – perinuclear anti-neutrophil cytoplasmatic antibody.

**Pattern/antibody**	**Hepatitis**	**Controls**	**p value**
pANCA	0	0	ns
Anti-actin	0	0	ns
ASMA	0	5.5% (4/73)	ns
AMA	0	5.5% (4/73)	ns
LKM	0	0	ns
ANA	27.3% (3/11)	31.5% (23/73)	ns
AC-2	0	4.1% (3/73)	ns
AC-3	0	1.4% (1/73)	ns
AC-4	18.2% (2/11)	8.2% (6/73)	ns
AC-8	0	4.1% (3/73)	ns
AC-12	0	0	ns
AC-13	0	1.4% (1/73)	ns
AC-25	0	1.4% (1/73)	ns
AC-27	0	1.4% (1/73)	ns
AC-15/16/17	0	1.4% (1/73)	ns
AC-18	0	2.7% (2/73)	ns
AC-19/20	0	12.3% (9/73)	ns
AC-23	9.1% (1/11)	1.4% (1/73)	ns
Total IB	27.3% (3/11)	16.4% (12/73)	ns
AMA-M2	0	4.1% (3/73)	ns
M2-E3	0	2.7% (2/73)	ns
Sp100	9.09% (1/11)	0	ns
gp210	0	0	ns
LC1	0	5.5% (4/73)	ns
Ro-52	18.2% (2/11)	6.8% (5/73)	ns
IgG	10.3 (8.1-12.6)	9.8 (9.2-10.5)	ns
