# Supplementary material for: Presence of autoantibodies in serum does not impact the occurrence of immune checkpoint inhibitor-induced hepatitis in a prospective cohort of cancer patients
Source: J Cancer Res Clin Oncol. 2021 Dec 7;148(3):647–56. doi: 10.1007/s00432-021-03870-6 (PMC8881258; doi:10.1007/s00432-021-03870-6)
Supplement: Supplementary file 7 — Supplementary file7 (PDF 370 KB) [file 432_2021_3870_MOESM7_ESM.pdf]

Supplementary Table 6. HLA alleles associated with autoimmune liver diseases and irAEs related to checkpoint inhibitor therapy in European populations. AIH – autoimmune hepatitis, HLA – human leukocyte antigen, irAE – immune-related adverse event, PBC – primary biliary cholangitis, PSC – primary sclerosing cholangitis.

**Locus**	**Allele**	**Association**	**References**
HLA-A	*01:01	AIH	[1]
HLA-B	*07:02	PBC (protective)	[1]
	*08:01	AIH, PSC	[1, 2]
	*39:05	PBC	[1]
HLA-C	*04:01	AIH	[3]
	*04:03	AIH	[3]
	*07:01	AIH	[4]
HLA-DPB1	*03:01	PBC	[5]
	*04:01	AIH	[4]
HLA-DQB1	*02:01	AIH, PSC	[1, 2]
	*03:01	AIH, PBC (protective), PSC (protective), colitis	[1, 2, 6]
	*03:02	PBC, PSC (protective)	[1, 2]
	*03:03	PSC (protective)	[1, 2]
	*04:01	PBC	[1]
	*04:02	PBC	[1]
	*05:03	PBC	[2]
	*06:01	PBC	[1]
	*06:02	PSC, AIH (protective), PBC (protective)	[1, 2, 7]
	*06:03	AIH, PSC	[2, 7]
	*06:04	PBC (protective)	[1]
HLA-DRB1	*01:01	PSC	[1]
	*03:01	AIH, PSC	[2, 5]
	*04:01	AIH, PSC (protective)	[2, 5]
	*04:04	PBC	[2]
	*04:05	PBC, arthritis	[1, 8]
	*07:01	PSC (protective)	[1]
	*08:01	PBC	[1, 2]
	*08:03	PBC	[1]
	*11:01	PBC (protective), PSC (protective), pruritus	[1, 2, 6]
	*11:04	PBC (protective)	[1]
	*13:01	AIH, PBC, PSC	[1, 5, 9]
	*13:02	PBC (protective)	[1, 5]
	*15:01	PSC, AIH (protective), PBC (protective)	[1, 2, 7]

[1] Arndtz K, Hirschfield GM. The Pathogenesis of Autoimmune Liver Disease. Digestive diseases. 2016;34:327-33.

[2] Mells GF, Kaser A, Karlsen TH. Novel insights into autoimmune liver diseases provided by genome-wide association studies. Journal of autoimmunity. 2013;46:41-54.

[3] Strettell MD, Thomson LJ, Donaldson PT, Bunce M, O'Neill CM, Williams R. HLA-C genes and susceptibility to type 1 autoimmune hepatitis. Hepatology. 1997;26:1023-6.

[4] Umemura T, Ota M. Genetic factors affect the etiology, clinical characteristics and outcome of autoimmune hepatitis. Clinical journal of gastroenterology. 2015;8:360-6.

[5] Webb GJ, Hirschfield GM. Genetics of autoimmune liver disease: a brief summary for clinicians. Digestive diseases. 2014;32:e1-6.

[6] Hasan Ali O, Berner F, Bomze D, Fassler M, Diem S, Cozzio A, et al. Human leukocyte antigen variation is associated with adverse events of checkpoint inhibitors. European journal of cancer. 2019;107:8-14.

[7] Donaldson PT. Genetics of liver disease: immunogenetics and disease pathogenesis. Gut. 2004;53:599-608.

[8] Cappelli LC, Dorak MT, Bettinotti MP, Bingham CO, Shah AA. Association of HLA-DRB1 shared epitope alleles and immune checkpoint inhibitor-induced inflammatory arthritis. Rheumatology. 2019;58:476-80.

[9] Karlsen TH, Chung BK. Genetic Risk and the Development of Autoimmune Liver Disease. Digestive diseases. 2015;33 Suppl 2:13-24.
